# A new survey tool for evaluating pandemic preparedness in health services

**DOI:** 10.1186/s12913-022-08067-2

**Published:** 2022-05-27

**Authors:** Nicole McGill, Jennifer Weller-Newton, Catherine Lees

**Affiliations:** 1 Department of Rural Health; Faculty of Medicine, Dentistry and Health Sciences, The University of Melbourne, 49 Graham St, Shepparton, Victoria Australia; 2 Department of Education, Training and Research, Echuca Regional Health, 226 Service St, Echuca, Victoria Australia

**Keywords:** Pandemic preparedness, Healthcare, Disaster readiness, Survey, COVID-19

## Abstract

**Background:**

Rapid decision-making with limited resources and prior research to draw upon posed challenges for health service leaders globally when preparing for COVID-19. How do health services prepare for a pandemic and evaluate if the preparation has been effective? This study aimed to explore health workers’ perceptions and knowledge regarding preparedness for COVID-19 at a regional health service in Australia.

**Methods:**

A 32-item online survey was developed to evaluate preparedness across five scales: 1) Clinical, 2) Communication, 3) Environment, 4) Human Resources, and 5) General Preparedness. Data were analyzed using parametric and non-parametric statistics and qualitative content analysis.

**Results:**

Ninety-three employees completed the survey, with most working in clinical roles (58.1%). Respondents largely felt the health service was well-prepared (84.0%) and they were personally prepared (74.4%) to respond to COVID-19. Clinical and communication scale scores varied by role type. Respondents faced personal risk and resource shortages impacted their sense of safety; others felt adequately supported.

**Conclusions:**

A coordinated “whole hospital response”, accessible and inclusive communication, education, adequate resourcing, and employee wellbeing supports are necessary when preparing health services for sentinel events. This survey tool offers health services an approach to evaluating pandemic preparation. Continued advocacy for resources and wellbeing needs of health workers is paramount in future preparations.

## Background

During sentinel events, like the global coronavirus (COVID-19) pandemic, there is rapidly changing information and quick decisions are essential. Health services responded to the COVID-19 pandemic by attempting to prepare their organizations and the workforce for outbreaks, surges in cases, and increased demand on existing services and the workforce. Preparedness, in the context of disasters, crises, or emergencies, has been referred to as “the ability of governments, professional response organizations, communities and individuals to anticipate and respond effectively to the impact of likely, imminent or current hazards, events or conditions” [1]. Preparation in health services involved provision of education, information, and resources such as personal protective equipment (PPE).

In early 2020, the COVID-19 pandemic was a new and unprecedented virus. There was little evidence for clinicians or health service leaders to draw from when making decisions in preparing health services. Some prior studies focused on influenza, avian influenza, and severe acute respiratory syndrome (SARS) pandemics [2–4]. Imai [3] explored perceptions of health service employees in seven tertiary hospitals in Japan regarding individual and institutional preparedness for a potential influenza pandemic. They found most hospitals lacked specific measures to cope with a pandemic. However, high levels of institutional preparedness predicted higher individual/personal preparedness, such as perception and recognition of preventative measures, and coping attitude to risk among the workforce [3]. Wong [4] surveyed health workers in public clinics and tertiary hospitals in Singapore regarding concerns and preparedness in an avian influenza pandemic. High rates of concern were reported regarding falling ill during an outbreak, yet more than 70% personally felt prepared and over 80% felt their workplaces were prepared [4]. A survey of general practitioners (GPs; *n* = 427) in the United Kingdom (UK) regarding their preparedness plans for influenza found more than half were aware of pandemic preparedness plans [2]. However, just a quarter of GPs felt their practices would be able to adequately respond to a pandemic [2]. GPs expressed concerns regarding information overload, a lack of clear guidance, insufficient resources, and inadequate capacity within the health system to effectively respond [2]. Confidence in the ability to provide advice and information to patients was identified as one aspect to include in measures of pandemic preparedness [2]. Researchers have also emphasized the importance of having well-planned guidelines, protocols, response plans, and training in place within health services in case of disasters [5–7]. All employees need to be aware of and skilled in implementing such workforce protocols. While some health workers may have prior experience to reflect on regarding infection control or management of other diseases/viruses in the past, many employees may not have worked in such situations before.

In the Australian context, at the beginning of the pandemic, there was a dearth of research in rural and regional Australian health services to inform decision-making. Despite the key roles undertaken by non-clinical support staff in health services, few studies have explored perceptions of health workers across all departments and role types. Most focus on nurses, doctors, and contexts such as emergency and critical care departments (e.g., [8–12]). It was unknown what impact the education and other preparation initiatives implemented early in the COVID-19 pandemic had on regional health workers’ knowledge regarding COVID-19 preventative measures and management, and perceptions of preparedness to respond to outbreaks at work.

## Aims

This research project investigated the knowledge and perceptions of preparedness for COVID-19 among health workers at an outer regional health service in Victoria, Australia. In doing so, it sought to explore:

1. Readiness of health workers and the health service to respond to a COVID-19 outbreak,
2. Usefulness of health service initiatives in preparing health workers and protecting them from a COVID-19 outbreak at work,
3. Knowledge and awareness of health workers regarding COVID-19 practices and procedures,
4. Potential differences in preparedness relating to role type (clinical vs non-clinical support), and
5. Education, training, and resource needs to enhance health workers’ readiness for a COVID-19 outbreak at work.

## Methods

A cross-sectional survey design was used to collect both quantitative and qualitative data from health workers at one outer regional health service. A survey enabled perspectives to be obtained from a large sample of respondents on a broad range of items in a time-efficient manner [13]. The study received ethical approval from The University of Melbourne Human Research Ethics Committee (Project ID: 2056975.1).

### Study context

The study was undertaken within a health service located in outer regional Victoria, Australia. The health service has approximately 850 employees and a high dependency unit, emergency department, medical and rehabilitation wards, and a variety of outpatient services (e.g., allied health, consulting suites). The health service is located on a state border and provides services to clients from two states. At the time the study was conducted, there had been no known COVID-19 positive patients in the health service.

### Participants

Ninety-three employees completed the online survey. Respondent demographic information is summarized in Table 1. Respondents were predominantly female (92.5%, *n* = 86) and most were aged between 41 and 60 years. More than half of respondents described their main/primary role as clinical (e.g., allied health, doctor, nurse; 58.1%, *n* = 54) and worked across 37 departments or wards. When asked to nominate their primary department, these were grouped into six main discipline categories, with nursing being the most common (e.g., emergency, aged care, maternity; 41.6%, *n* = 32).

**Table 1:** Respondent demographics

		Number of responses *n* (%)	Valid *n*
Gender	Female	86 (92.5%)	93
	Male	4 (4.3%)	
	Prefer not to say/other	3 (3.2%)	
Age	Under 21 years	0 (0.0%)	93
	21-30 years	9 (9.7%)	
	31-40 years	14 (15.1%)	
	41-50 years	30 (32.3%)	
	51-60 years	34 (36.6%)	
	61 years and over	6 (6.5%)	
Education level	Up to year 10	1 (1.1%)	93
	Year 11	4 (4.3%)	
	Year 12	3 (3.2%)	
	TAFE certificate	10 (10.8%)	
	Graduate Diploma/ Certificate	19 (20.4%)	
	Bachelor degree	30 (32.3%)	
	Postgraduate qualification (e.g., Masters, PhD)	26 (28.0%)	
Primary role type	Clinical	54 (58.1%)	93
	Non-clinical support	38 (40.9%)	
	Both clinical and non-clinical	1 (1.1%)	
Primary department	Nursing	32 (41.6%)	77
	Administration	19 (24.7%)	
	Allied health	11 (14.3%)	
	Community services	8 (10.4%)	
	Corporate services	5 (6.5%)	
	Medical	2 (2.6%)	

### Instrument

A 32-item online survey was used, incorporating items from previously validated international pandemic survey tools [2–4] adapted to suit this study population. Likert scales used in the prior studies were modified from six to five response options (strongly disagree, disagree, undecided/not sure, agree, strongly agree) for the current study. “Not applicable” was also included as a response option, recognizing that some questions may not be relevant to all respondents’ roles. Both closed and open-ended questions were included. The survey was piloted on five health workers who held nursing, infection control, administration, human resources, engineering, and allied health roles, which led to minor revisions to the survey prior to distribution (e.g., useability, accessibility of embedded URLs within project information, delineation between discipline/department categories).

Survey items explored respondents’ views regarding general preparedness (e.g., how well-prepared they felt) and their perspectives and knowledge relating to the health service’s preparation initiatives/strategies, for instance, regarding how useful the strategies were in preparing them for or protecting them against a COVID-19 outbreak at work. Items evaluating similar aspects of preparation were grouped together to form five scales, with scale reliability calculated using Cronbach alpha: 1) Clinical, α = .861; 2) Communication, α = .778; 3) Environment, α = .795; 4) Human Resources, α = .642; and 5) General Preparedness, α = .710 (see Table 2). Scale reliability for the combined scales was high (α = .899) [14]. An overview of items within each scale is outlined as follows (also see Table 2).

**Table 2:** Preparedness for COVID-19 survey scale items and responses

			Agreement (agree/ strongly agree responses)	
Scale	Question	Item	Number (%)	Valid *n*
1. Clinical	a. Do you believe that the following clinical measures are useful in protecting you from contracting/being infected with coronavirus (COVID-19) at work?	i. Hand washing [3]	85 (97.7%)	87
		ii. Alcohol rubs/hand sanitizer [3]	85 (96.6%)	88
		iii. Surgical mask (face mask) [3]	66 (83.5%)	79
		iv. N95 mask (respirator mask) [3]	67 (87.0%)	77
		v. Gloves [3]	73 (89.0%)	82
		vi. Gowns [3]	77 (96.3%)	80
		vii. Goggles [3]	71 (89.9%)	79
		viii. Antiviral drugs [3]	10 (14.1%)	71
	b. Please indicate your level of agreement with the following statements:	i. There is an infection control committee at my hospital [4]	82 (93.2%)	88
		ii. I have been recommended by the hospital to receive a flu vaccination (‘flu shot’) [3, 4]	88 (100.0%)	88
		iii. There are infection control staff in my hospital [4]	87 (98.9%)	88
		iv. My hospital has a plan for a coronavirus (COVID-19) outbreak [2–4]	80 (90.9%)	88
		v. My hospital has developed/adopted clinical guidelines for responding to a coronavirus (COVID-19) outbreak	81 (93.1%)	87
	c. Please indicate your level of agreement with the following statements. In the past 3 months:	i. I have attended infection control training sessions [4]	58 (73.4%)	79
		ii. I have participated in infection control audits [4]	20 (27.4%)	73
		iii. I have attended infection control related meetings [4]	32 (43.2%)	74
		iv. I have received a flu vaccination (‘flu shot’) [3, 4]	83 (98.8%)	84
		v. I have someone to turn to if I have a problem in using personal protective equipment (PPE) [3, 4]	70 (90.9%)	77
2. Communication	a. Do you believe that the following communication strategies are useful in preparing you to respond to coronavirus (COVID-19) at work?	i. Information posters/notices [3]	76 (89.4%)	85
		ii. COVID-19 email updates	83 (98.8%)	84
		iii. Video updates from the CEO regarding coronavirus (COVID-19)	61 (72.6%)	84
		iv. Information on the health service intranet regarding coronavirus (COVID-19)	76 (89.4%)	85
		v. Health service social media updates regarding coronavirus (COVID-19)	65 (79.3%)	82
		vi. Updates from the health service Department Heads regarding coronavirus (COVID-19)	76 (91.6%)	83
		vii. Meetings discussing coronavirus (COVID-19)	73 (89.0%)	82
		viii. Downloading the COVIDSafe app	52 (62.7%)	83
3. Environment	a. Do you believe that the following environmental strategies are useful in protecting you from contracting/being infected with coronavirus (COVID-19) at work?	i. Limiting visitors to the health service [3]	79 (97.5%)	81
		ii. Temperature checks (for patients and visitors) [3]	76 (92.7%)	82
		iii. Social distancing at work	75 (91.5%)	82
		iv. Limiting shared desks/workstations (“hot-desks”)	73 (90.1%)	81
		v. Area isolation (Restricting employee/visitor access to areas) [3]	78 (95.1%)	82
		vi. Increasing airflow through work areas	56 (68.3%)	82
		vii. Additional cleaning/sanitization of work areas/offices	78 (96.3%)	81
		viii. Reduced frequency of work meetings	70 (86.4%)	81
		ix. Telehealth sessions (limiting face-to-face work with patients)	76 (98.7%)	77
		x. Limiting clinical placements at the health service	61 (79.2%)	77
4. Human Resources	a. Do you believe that the following human resources related strategies are useful in supporting you/protecting you from coronavirus (COVID-19) at work?	i. Temperature checks (for employees) [3]	75 (87.2%)	86
		ii. Asymptomatic COVID-19 screening tests for employees	76 (88.4%)	86
		iii. Employees working from home	68 (81.9%)	83
		iv. Flexible leave planning	73 (87.9%)	83
	b. Please indicate your level of agreement with the following statements:	i. I am aware of the health service’s COVID-19 Health and Wellbeing program for employees	70 (82.4%)	85
		ii. I have accessed the health service’s COVID-19 Health and Wellbeing program for employees	16 (21.6%)	74
		iii. I found it helpful to access the health service’s COVID-19 Health and Wellbeing program for employees	16 (26.7%)	60
5. General Preparedness	a. Please indicate your level of agreement with the following statements:	i. My hospital is well prepared to respond to a coronavirus (COVID-19) outbreak [2–4]	68 (84.0%)	81
		ii. I have been involved in decision-making regarding coronavirus (COVID-19) preparation in my role/area at the health service	42 (57.5%)	73
		iii. I am well prepared to respond to a coronavirus (COVID-19) outbreak in my role at the health service [4]	58 (74.4%)	78
		iv. I am confident in explaining coronavirus (COVID-19) procedures to colleagues at work	63 (84.0%)	75
		v. I am confident in explaining coronavirus (COVID-19) pandemic to patients [4]	58 (82.8%)	70
		vi. I am confident in explaining coronavirus (COVID-19) pandemic to members of the community	61 (82.4%)	74
		vii. I believe decisions regarding coronavirus (COVID-19) preparation at the health service were based on the best available evidence at the time	72 (88.9%)	81
		viii. I believe the scale of the health service’s response to coronavirus (COVID-19) preparation was appropriate for the level of potential risk/threat	66 (81.5%)	81
		ix. I have personally coped with the threat of a coronavirus (COVID-19) outbreak at work by learning as much as I can about it [3]	71 (88.8%)	80
		x. I would accept the risk of contracting coronavirus (COVID-19) at work in the event of an outbreak [3, 4]	49 (60.5%)	81
		xi. I am afraid of falling ill with coronavirus (COVID-19) in the event of an outbreak at work [3, 4]	40 (50.0%)	80
		xii. I am worried about a second wave of coronavirus (COVID-19) outbreak at work	54 (66.7%)	81
		xiii. I might consider taking extended leave from my role because of the risk of contracting coronavirus (COVID-19) at work [3]	13 (16.3%)	80
		xiv. I might look for another job or consider resigning because of the risk of contracting coronavirus (COVID-19) at work [3, 4]	1 (1.3%)	79
		xv. My colleagues should stay home from work if they have cold/flu symptoms during the coronavirus (COVID-19) pandemic	77 (97.5%)	79
		xvi. I feel it is pointless to take precautions regarding coronavirus (COVID-19) at work [3]	1 (1.2%)	81

Items adapted from: [2–4]

Specific name of the study site has been replaced with generic term “health service” where applicable

#### Clinical scale

This scale comprised 18 items which measured respondents’ knowledge and awareness regarding infection control and other clinical initiatives for COVID-19 preparation. Respondents were asked to indicate their level of agreement regarding the usefulness of clinical measures (e.g., hand washing, masks, hand sanitizer) in protecting against COVID-19, and their knowledge of and participation in infection control initiatives and planning.

#### Communication scale

Eight items were included in this scale to evaluate the pandemic communication strategies implemented by the health service. Respondents indicated their level of agreement regarding the usefulness of each communication strategy/initiative (e.g., COVID-19 email updates, information posters) in preparing them for a COVID-19 outbreak at work.

#### Environment scale

This scale comprised 10 items and respondents were asked to rate their level of agreement regarding the usefulness of environmental strategies implemented in the health service (e.g., limiting visitors, shared desks, and student placements) for protecting them against COVID-19 infection.

#### Human resources scale

The seven items in this scale evaluated the usefulness of the health service’s human resources strategies (e.g., working from home) in supporting and protecting respondents from COVID-19 infection, and respondents’ awareness and perspectives regarding the COVID-19 Health and Wellbeing program offered in the health service.

#### General preparedness scale

This scale comprised 16 items which evaluated the health service’s overall preparation and individual respondents’ level of readiness. Respondents were asked about their confidence in explaining COVID-19 information and perspectives regarding decision-making, level of fear and worry, and beliefs regarding COVID-19 precautions.

### Procedure

#### Recruitment

All employees within the health service (approximate *n* = 850) working in all wards and departments were invited to participate, including those in clinical and non-clinical support roles. Recruitment occurred over a two-week period in June 2020, during the ‘first wave’ of COVID-19 in Australia. The survey was distributed via email invitations containing the online questionnaire URL to department heads and all staff/organization-wide mailing lists, in COVID-19 email updates circulated organization-wide three times per week, as well as by word of mouth. Reminder emails were distributed after 1 week via the same communication channels as the initial invitation, then the survey was closed at the end of the second week of recruitment.

#### Data collection

Upon accessing the survey URL, respondents viewed information about the study and ethical approval. They were asked to respond to a mandatory question indicating they had understood the information and that proceeding with the survey indicated their consent to participate in the research.

#### Data analysis

Respondents’ answers to closed questions were collated in the online survey program and exported into IBM SPSSv26 [15] for data cleaning and analysis. Descriptive statistics and frequency analyses were undertaken to address research aims one to three. Respondents answered multiple choice questions and indicated their level of agreement with a range of statements on a scale of 1 (strongly disagree) to 5 (strongly agree). “Agree” and “strongly agree” responses were combined for each item and considered agreement when reporting the findings. “Not applicable” responses were treated as missing data and excluded from these analyses.

Analysis was also conducted on total scale scores to address research questions one to four. Five items within the General Preparedness scale were negatively worded and were reverse coded during data cleaning. Respondents who had more than one missing data point (no response provided) within a scale were excluded from the analysis to avoid skewing the results. Respondents’ scores on each scale between role types (clinical versus non-clinical) were compared using independent samples t-tests for variables meeting the assumption of normality, while those violating the assumption of normality were analyzed using the non-parametric alternative of Mann-Whitney U Test. For those analyses, one respondent who indicated they worked in both clinical and non-clinical support roles was excluded to enable sufficient sample sizes and maintain data integrity for the two groups.

Qualitative content analysis was undertaken on open-ended comments to address research questions one, two, and five. This entailed a four-stage process of: i) decontextualization – identifying meaning units/codes, ii) recontextualization – excluding extraneous content, iii) categorization – identifying homogenous groups, this process of triangulation was undertaken by two of the researchers, and iv) compilation, drawing conclusions through staying close to respondents’ text [16]. Respondents were given a unique code number, and this is referred to in reporting the qualitative findings.

## Results

Ninety-three completed surveys were analyzed, indicating an 11% response rate, and the results are presented below.

### COVID-19 education and training

Respondents participated in a range of health service education and training initiatives focused on COVID-19 procedures and patient care (Table 3). Over a third participated in online training offered by external organizations (38.7%, *n* = 36). Six respondents (6.5%) participated in other forms of education, such as COVID-19-specific self-care/palliative end-of-life-care pathways, daily COVID-19 update meetings, reading journal articles, and accessing the health service’s repository of policies and procedures. Just six respondents (6.5%) had not yet participated in any education or training sessions about COVID-19.

**Table 3:** Respondents’ participation in COVID-19 education^a^ (*N* = 93)

COVID-19 related education	Number of responses *n* (%)
Online hand hygiene eLearning	80 (86.0%)
Donning and doffing personal protective equipment (PPE)	69 (74.2%)
Online COVID-19 infection control training eLearning	63 (67.7%)
Consultation with infection prevention and control staff at the health service	51 (54.8%)
Webinars/online training offered by external organizations	36 (38.7%)
‘Short and sharp’ COVID-19 simulation	20 (21.5%)
Simulation – airway management for intubation specific to COVID-19 patients	14 (15.1%)
Simulation – advanced life support for the COVID-19 patient	12 (12.9%)
Attended other education or training	6 (6.5%)
Did not attend any education or training sessions about COVID-19	6 (6.5%)

^a^ Respondents could select more than one response

### Perspectives and knowledge regarding health service preparation

Respondents were also asked about their perspectives and knowledge regarding COVID-19 preparation initiatives and strategies in the health service, in a series of items within five scales (Table 2). Handwashing (97.7%, *n* = 85), alcohol rubs/sanitizer (96.6%, *n* = 85), and gowns (96.3%, *n* = 77) were considered the most useful clinical measures for protecting against COVID-19 infection by the respondents. All respondents had been recommended to get a flu vaccination and almost all had followed this recommendation (98.8%, *n* = 83). Over 90% of respondents were aware there was an infection control committee and infection control staff at the health service, and that there was a plan in place for a COVID-19 outbreak. Organization-wide COVID-19 email updates (99%, *n* = 83) and updates from department heads (92%, *n* = 76) were felt to be the most useful communication strategies implemented to prepare employees for COVID-19. Limiting face-to-face work with patients during the pandemic (99%, *n* = 76), limiting visitors to the health service (98%, *n* = 79), and additional cleaning of work areas (96%, *n* = 78) were perceived to be the most useful environmental strategies. Asymptomatic COVID-19 screening tests for employees (88.4%, *n* = 76) were seen as the most useful human resources strategy for supporting/protecting respondents from infection, followed by flexible leave planning (87.9%, *n* = 73) and temperature checking of employees (87.2%, *n* = 75). Over 80% of respondents were aware there was a health and wellbeing program available to employees at the health service.

Regarding general preparedness, most respondents agreed or strongly agreed that they were ready to respond to a COVID-19 outbreak in their role (74.4%, *n* = 58) and that the health service was well-prepared overall (84.0%, *n* = 68). Decisions regarding preparation of the health service were thought to be based on the best available evidence at the time (88.9%, *n* = 72) and the scale of response was appropriate for the level of potential risk/threat posed (81.5%, *n* = 66). Many respondents had coped with the threat of an outbreak at work by learning as much as possible about it (88.8%, *n* = 71). Two-thirds of respondents were worried about a second wave of COVID-19 (66.7%, *n* = 54) and half were afraid of falling ill with COVID-19 at work (*n* = 40, 50.0%). However, very few respondents considered taking extended leave or looking for another job due to the risk of contracting COVID-19 at work. Sixty percent (*n* = 49) indicated they would accept the risk of contracting the virus at work in the event of an outbreak.

### Differences in employees’ perspectives and knowledge

Respondents’ scores on each of the five scales were compared to ascertain any differences across clinical and non-clinical groups and findings are reported separately for each scale.

#### Clinical scale

Mann-Whitney U Test identified a statistically significant difference in scores for clinical (*Md* = 78.00, *n* = 46) and non-clinical (*Md* = 70.00, *n* = 37) workers with a medium effect size, *U* = 555.50, *z* = − 2.710, *p* = .007, *r* = .30 [17].

#### Communication scale

Mann-Whitney U Test identified a statistically significant difference between clinical (*Md* = 35.0, *n* = 48) and non-clinical (*Md* = 31.0, *n* = 36) workers’ perspectives regarding health service communications, with a small to medium effect size, *U* = 604.50, *z* = − 2.352, *p* = .019, *r* = .26 [17].

#### Environment scale

Mann-Whitney U Test found no significant difference in total scores for clinical (*Md* = 45.0, *n* = 45) and non-clinical workers (*Md* = 44.0, *n* = 36), *U* = 723.50, z = −.827, *p* = .408, *r* = .09.

#### Human resources scale

An independent samples t-test identified no significant difference in scores for clinical (*M* = 25.33, *SD* = 4.24, *n* = 48) and non-clinical workers (*M* = 24.57, *SD* = 5.47, *n* = 37; *t*(83) = .73, *p* = .469, two-tailed).

#### General preparedness scale

An independent samples t-test found no significant difference in scores for clinical (*M* = 61.95, *SD* = 7.12, *n* = 44) and non-clinical workers (*M* = 57.47, *SD* = 12.05, *n* = 36; *t*(54.18) = 1.97, *p* = .054, two-tailed).

### Health workers’ perceptions

Four key themes were inductively identified from the respondents’ free text responses that encapsulated their perceptions of preparedness: leadership, resources, knowledge acquisition, and feeling prepared and supported.

#### Leadership

There were mixed perspectives regarding leadership and management decisions within the health service. Comments related to communication patterns and processes, response coordination, and equity and inclusiveness across departments. Respondents’ views varied regarding the amount, format, and style of COVID-19-related communications. Department- and ward-specific communication delivered by managers “*in the initial phase of the COVID outbreak was really helpful including the emails and the COVID communication book”* (77). Suggestions were made regarding alternate avenues of information via SMS to staff or at strategic team meetings. Some respondents considered the information and communications to be excessive; one respondent felt “*the updates need to be short and sharp, not the waffle we sometimes got*” (50) and another reported there were “*too many signs which turns into ‘visual noise*’ *and people begin to ignore them all*” (88).

Several ways in which leadership could enhance coordination and communication were recommended by respondents, ranging from having a “*multidisciplinary approach to communicating and planning”* (77) to ensuring consistency in all elements and across all wards/areas. Having a central point for information or “*one person as a main contact/disseminator of advice*” (12) was also recommended along with ensuring the pandemic response was coordinated with other local partner organizations and health services *“to ensure we are working together, partnering where possible and being consistent in our message and support to one another”* (10).

Involving and respecting all health workers throughout the organization was emphasized by respondents regarding decision-making, communication, and resourcing. Respondents identified the need for “*crisis management planning inclusive of all areas not just frontline*” (27) along with providing “*ALL staff with protection*” (40), “*not just in what are perceived to be … ‘clinical areas’*” (5).

#### Resources

Respondents' comments predominantly related to protective measures, staffing, and cleaning. Common concerns were personal risk and difficulties obtaining resources to enable them to feel protected and prepared, and safely care for clients. One respondent was particularly concerned about the lack of resources and how this could impact on their care delivery:

“*lack of PPE and hand sanitizers/wipes have been a concern affecting our ability to provide care to clients … I am prepared to accept risk of COVID-19 IF we have adequate PPE to use. I would be unhappy about having to see clients when PPE are in limited supply … with no hand sanitizers in some treatment rooms, I would have to weigh* *[up]* *if I would see clients”* (8).

Rationing the limited PPE supplies reportedly “*increased the stress level to be able to perform your job*” (36). Employing extra cleaning staff and “*implementing a formal back fill process for administrative/reception staff*” (51) were suggestions to assist with increased demands.

#### Knowledge acquisition

Respondents described the need for further guidelines and training to inform their work and enhance their sense of preparedness. This related to augmenting clinical skills in recognizing symptoms of COVID-19 including simulations of “*mock scenarios dealing with patients having the virus*” (73) and implementing protocols and guidelines. Further education and training on the use of appropriate PPE was suggested along with having,

“*designated time and coursework initiated by senior staff and the medical team to help understand how to be prepared* *[and]* *understand our individual roles”* (73).

Education and implementation of infection control protocols and guidelines were also mentioned including isolation protocols, office spacing, and prevention of transmission.

#### Feeling prepared and supported

Many respondents reported a general sense of preparedness and reflected upon and valued the efforts of their colleagues, management, and the health service as indicated below,

*“management and staff have prepared well and have adequate support”* (42)

*“our organization has responded really well and been led effectively to be prepared, but not alarmed as we navigated this virus”* (27).

*“I feel have been well educated and trained re: COVID-19”* (10).

However, some respondents described negative impacts on their wellbeing and performance during what was felt to be a *“a scary time as no-one knew how the virus would escalate*” (30). The issue of limited resources and staff shortages impacted on their levels of stress as one respondent commented, “*rations per shift* *[which]* *increased the stress level to be able to perform your job”* (36). Others wanted to be better informed about aspects of COVID-19 and PPE, for instance “*more information and continuity as* *[to]* *when and what* *[PPE]* *to wear”* (75).

This analysis of respondents’ comments adds depth to the understanding of health workers’ perceptions of the pandemic preparation and education they received.

## Discussion

The findings of this survey support the notion that this regional health service’s initiatives to prepare for COVID-19 had been useful and effective, with most health workers indicating their satisfaction with the level of preparedness. The health service initiatives implemented may inform planning and preparation in other similar health services in future sentinel events. The survey developed in this study may be a useful tool for other health services to evaluate the preparation and education of their workforce.

Health workers face a degree of personal risk of becoming infected and falling ill at work during the COVID-19 pandemic. Thirty-eight percent indicated they would not accept the risk of contracting the virus, slightly higher than reported for health workers in Singapore regarding risk of contracting avian influenza [4]. However, consistent with the findings of Wong [4], few indicated they would resign from their jobs due to the risk. Given that the pandemic has continued and extended its reach with new variants of the virus, the safety and wellbeing of the health workforce has become increasingly important to sustain. Fear and worry among health workers may stem from a range of factors other than the risk of COVID-19 relating to themselves or their family, such as their overall health status or pre-existing mental health issues, potentially requiring these factors to be considered when allocating roles and shifts during pandemics [10]. Health workers’ fear has been reported in other studies, including research involving frontline nurses [10]. Labrague [10] recommended that measures be in place within health services to support health workers’ mental health and address fear of COVID-19, including “peer and social support, psychological and mental support services (e.g., counselling or psychotherapy), provision of training related to COVID-19 and accurate and regular information updates”. Although peer and social support were not explored in the present study, COVID-19 training and education, daily information updates, and mental health and wellbeing services for health workers were implemented at the study site. Attending COVID-19-related training and accessing information are associated with decreased levels of fear among frontline health workers [10]. Yet, some respondents in the present study described their need for additional support, training, information, and resources to feel prepared. Shortages of resources such as PPE contributed to some respondents’ sense of feeling unsupported and unprotected.

The issue of finite resources that were initially unable to meet the increased needs of the health service posed ethical dilemmas. Difficult decisions needed to be made by leadership and management teams regarding distribution and rationing of limited resources to minimize risk and prioritize the allocation of resources, for instance, based on frontline status or perceived vulnerability of employees or departments. Health service managers play a key role in preparation for future pandemics by advocating for resources and ensuring the availability and replacement of quality, effective PPE to enhance employees’ sense of safety and minimize risk of infection [8]. Further workforce education and training are needed to ensure staff are abreast of the evolving nature of pandemics. In Australia, inroads have been made regarding accessibility of mental health and wellbeing support services for health workers as the pandemic extended, including government-funded rebates for telehealth services. However, continued efforts should be made to ensure those undertaking shift work and working on the frontline in under-resourced departments are catered for, as they may require additional flexibility in service delivery. The fear, worry, sense of risk, and support needs of the workforce highlighted in this study emphasize the need to consider these areas in future pandemic preparation and in the evaluation of health service initiatives.

The differences in clinical scale scores regarding role type may relate to various factors. Clinical staff probably had greater knowledge of and participated in clinical initiatives regarding COVID-19 relating to the requirements of their roles. Some of the clinical scale items were more likely to be relevant for clinical staff directly involved in patient care. The largest proportion of respondents worked in nursing departments and since nurses receive infection control training and are generally more involved in direct infection control care than many other disciplines, this may have influenced their perceptions [3]. Differences between groups may also have related to the amount of previous exposure to infection control management of other viruses (e.g., influenza); differences in skillsets and ability to locate, understand, interpret, and apply COVID-19 information and integrate it into decision-making; varied levels of relevance of and engagement in the education and training offered; or differing involvement in decision-making and management regarding COVID-19 preparation. An awareness of infection control processes and protective equipment has become increasingly important in health services and broader community contexts as the pandemic evolves. Enhanced preparation across all levels of health services, including in non-clinical areas, may be beneficial for future pandemics [3].

Most COVID-19 related information in the health service was communicated via emails or the intranet and some employees in non-clinical support roles (e.g., corporate services) at the study site had limited access to computers, potentially contributing to the differences in responses on the communication scale. A more coordinated *“whole hospital response”* was recommended when communicating COVID-19 related messages and information. Information overload and difficulty keeping up to date with rapidly changing information from multiple sources has similarly been reported in prior studies (e.g., [11, 12]). The challenges of “inconsistent messaging and lack of clear communications from management” have also been reported by Australian emergency clinicians during the COVID-19 pandemic [11]. Hospital information needs to be streamed, concise and relevant [18]. Hence, health services are encouraged to keep terminology simple and consider alternative communication strategies. This could include centralizing sources of information and enhancing coordination of organization-wide communications, whilst ensuring communications are accessible for all employees with diverse educational backgrounds and role types (e.g., through use of infographics). This is key to achieving a coordinated pandemic response.

### Limitations

The present study explored perspectives of employees from one health service in outer regional Victoria, Australia, which may impact the applicability of the findings beyond this context. Given there were no active COVID-19-positive cases at the health service at the time of survey distribution, preparedness may have been overestimated by respondents. Most respondents were female nurses. Although nursing is the largest proportion of the health workforce and is a female dominated profession, this may limit generalizability of findings to male employees and those working in other departments. The low response rate for the survey overall may also impact generalizability. It is not possible to attribute respondents’ knowledge, confidence, and perceptions of preparedness solely to the health service’s initiatives since some accessed information and training external to the health service and likely worked in more than one organization. Health workers’ preparedness may also have been impacted by factors other than role type (e.g., demographics) not analysed in this paper. Additionally, some survey items measured employees’ perceptions of the usefulness of strategies and initiatives implemented by the health service in preparing/protecting them from COVID-19 outbreaks, not necessarily how prepared respondents felt in relation to each domain. However, since the preparedness of the organization may predict individual preparedness [3] and each scale contributed to evaluating the health service’s multifaceted preparation for COVID-19, this was deemed appropriate.

### Future directions

The present study was conducted in the early stages of the pandemic in 2020, around the time of the first lockdowns in Victoria, Australia, and learnings from other health services were then implemented, more environmental strategies were put in place, and PPE supplies increased. Further research using this evaluative survey tool is needed to validate the tool for use in other contexts, including in other health services, with larger sample sizes to enable factor analysis to be conducted and use of Omega to measure scale reliability. Exploration of demographic factors impacting health workers’ preparedness may also provide insights to guide health service preparation and inform future iterations of the survey tool. Although survey items were tailored for COVID-19, they may be adapted for other disaster or pandemic situations to evaluate preparation of health services.

## Conclusion

Preparation of health services during pandemic or crisis situations is a fine balance between transparency, equity, and understanding health workers’ concerns regarding personal risk. Hence, communication and educational strategies need to reflect the diversity of educational backgrounds and role types of health workers. The survey tool used in this study provides one way of evaluating health service preparation. There is an ongoing need to monitor and maintain the readiness of the health service workforce in responding to the COVID-19 pandemic, plan for recovery, and prepare for future sentinel events.

## Data Availability

The datasets used and/or analyzed during the current study are included in this published article.
